# Perceptions of pregnancy occurring among HIV-serodiscordant couples in Kenya

**DOI:** 10.1186/s12978-019-0751-4

**Published:** 2019-06-18

**Authors:** Grace Kimemia, Kenneth Ngure, Jared M. Baeten, Connie Celum, Kristin Dew, Njambi Njuguna, Nelly Mugo, Renee Heffron

**Affiliations:** 1 African Population & Health Research Center, Nairobi, Kenya; 2 Department of Community Health, Jomo Kenyatta University of Agriculture and Technology, Nairobi, Kenya; 3 Department of Global Health, University of Washington, 325 Ninth Avenue Box 359927, Seattle, WA 98104 USA; 4 Department of Epidemiology, University of Washington, 325 Ninth Avenue Box 359927, Seattle, WA 98104 USA; 5 Department of Medicine, University of Washington, Seattle, USA; 6 Department of Human Centered Design and Engineering, University of Washington, Seattle, USA; 7 FHI360, Nairobi, Kenya; 8 Center for Clinical Research, Kenya Medical Research Institute, Nairobi, Kenya

**Keywords:** safer conception, perceptions, HIV prevention, Pregnancy

## Abstract

**Background:**

Among HIV serodiscordant couples, most conception involves condomless sex and may confer a period with increased HIV transmission risk if HIV viral load is not suppressed and other precautions are not used. Safer conception strategies enable HIV serodiscordant couples to attain their pregnancy goals while markedly reducing this risk. We explored the perceptions and beliefs held by HIV serodiscordant couples and health care providers concerning pregnancy among HIV serodiscordant couples in Kenya and gathered their thoughts about how these might influence use of safer conception methods.

**Methods:**

We conducted 20 Key Informant Interviews (KIIs) with health care providers offering safer conception counseling and 21 In-Depth Interviews (IDIs) and 4 Focus Group Discussions (FGDs) with members of HIV serodiscordant couples with immediate pregnancy goals in Thika, Kenya. Data were analyzed using an inductive approach that identified two emergent themes: perceptions towards pregnancy among HIV serodiscordant couples and access to safer conception services.

**Results:**

The perceptions held by the community towards couples in HIV serodiscordant relationships having children were largely negative. The participants were aware of the increased HIV transmission risk to the HIV uninfected partners while trying to become pregnant. In the community, having biological children was cherished yet the majority of the couples shied away from accessing safer conception services offered at health facilities due to stigma and lack of knowledge of the existence of such services. Some providers had limited knowledge on safer conception strategies and services and consequently discouraged HIV serodiscordant couples from natural conception.

**Conclusions:**

Negative perceptions towards HIV serodiscordant couples becoming pregnant has hindered access to safer conception services. Therefore, there is need to create a supportive environment for HIV serodiscordant couples with fertility intentions that normalizes their desire to have children and informs the community about the availability of safer conception services.

## Plain English Summary

When HIV serodiscordant couples try to become pregnant, the chance that the partner with HIV will pass their infection to their partner becomes bigger. Safer conception strategies can be used by couples to lower the chance that the HIV is passed during pregnancy tries. We explored ways that people think about pregnancy when it occurs among people affected by HIV and how these ideas could influence whether a couple uses safer conception strategies.

We individually interviewed 20 health care providers and 21 members of HIV serodiscordant couples and we held discussions with 4 groups of members of HIV serodiscordant couples. The members of HIV serodiscordant couples had expressed their current goal to become pregnant. We analyzed the data to explore their perceptions towards pregnancy among HIV serodiscordant couples and if these perceptions influence whether people could receive safer conception services.

We found that the community has negative ideas towards people living with HIV having children. People assumed that HIV would be passed to the negative partner when trying to become pregnant. Most couples were shy about coming to the clinic because they feared that they would be made to feel ashamed. Health providers did not always know all of the safer conception techniques and were not always able to support the couples.

It is important to create a supportive environment for HIV serodiscordant couples to realize their goals for building a family and to educate communities about safer conception so that they also support couples.

## Background

In Kenya, over forty percent of HIV infected individuals have HIV uninfected regular partners [1]. Many of these couples are of reproductive age and have strong desires to have children, which may put the HIV uninfected partner at risk of sexual HIV acquisition as they pursue their pregnancy goals via condomless sex [2]. Safer conception strategies offer HIV serodiscordant couples an opportunity to conceive while reducing the risk of sexual transmission [3]. These strategies include antiretroviral therapy (ART) by HIV infected partners for viral suppression, pre-exposure prophylaxis (PrEP) use by the HIV uninfected partner [3–7], restricting condomless sex to peak fertility [8, 9], vaginal self-insemination for couples with HIV infected women [10], male circumcision for HIV uninfected men [11, 12], and medically assisted reproduction [13, 14]. When used singly or in combination, these interventions reduce the risk of HIV transmission while permitting pregnancy, and are recommended by international and Kenyan HIV prevention guidelines [15, 16].

Small-scale safer conception demonstration programs, including ones in Africa, have recently observed great success with healthy conceptions and pregnancies without sexual HIV transmission [17] and many are calling for safer conception programs to be brought to scale [18–20]. A community environment that supports HIV serodiscordant couples to have children would greatly facilitate demand for safer conception services and promote assessment of fertility intentions and the incorporation of safer conception services into HIV programs [21, 22]. However, in many communities, people living with HIV have historically been discouraged by health care workers from becoming pregnant [23]. This is due to the risk of sexual and perinatal HIV transmission as well as lingering concerns about HIV-associated mortality and whether HIV-infected parents will live to raise children through to adulthood [24]. More than three decades into the HIV epidemic, those concerns are largely mitigated through well-tolerated and highly effective ART which is now recommended at all CD4 counts [25]. The impact of evolving ART guidelines, stigmatization in the community, and provider knowledge about safe conception in African settings with high HIV prevalence and fertility is unknown. With this study, we aimed to explore the prevailing types of stigma and discrimination that HIV serodiscordant couples experience when trying to have children in Kenya.

## Methods

### Study design and population

To meet our objectives to understand attitudes held in the community, we conducted one-time qualitative interviews in Thika and Nairobi, Kenya between August 2015 and March 2016. We conducted Key Informant Interviews (KIIs) with health care providers working with HIV serodiscordant couples desiring to have children and in depth interviews (IDIs) and focus group discussions (FGDs) with members of HIV serodiscordant couples. At the point of data saturation, we had conducted 21 in-depth interviews with members of HIV serodiscordant couples and 4 FGDs with a total of 25 participants stratified by gender and HIV status. Ten people were invited per FGD and the discussion was conducted if at least five attended. The first FGD included 8 HIV positive women, the second included 5 HIV negative men, the third included 6 HIV positive men and the last included 6 HIV positive women.

This study was nested within the Partners Demonstration Project, an open-label evaluation of an integrated delivery strategy for PrEP and ART to 1,013 high-risk HIV serodiscordant couples which demonstrated the virtual elimination of HIV transmission risk (<0.5% per year) [4]. During the quarterly visits, all couples participating in the Partners Demonstration Project at the Thika, Kenya site who expressed their fertility desire to have a child immediately or in the near future were invited and scheduled to participate individually for either IDIs or FGD at a time and place of their convenience [26]. For FGD, people living with HIV and HIV-negative people were invited on separate days to participate in the interview. However, for IDIs the couple was invited to participate in the interviews but each individual was consented and interviewed separately. Interviewing individual members of the couple for IDI separately brought the interviewer closer to the unhindered voice of the participant.

In addition, we approached health care providers in Nairobi and Thika working at public and private fertility clinics, public health hospital comprehensive care clinics (CCC) and safer conception research sites. Most of these providers were known to the study team through their engagement as referral partners for participants in the Partners Demonstration Project who needed specialized care beyond the services available at the research clinic. In addition to serving as study participants, the health care providers could refer their colleagues working with HIV serodiscordant couples to the study recruitment team so that they may also participate in the study.

### Data Collection

All interviews were conducted by an experienced social scientist who underwent a one-week protocol-specific training. The training included reviewing the informed consent, the semi-structured IDI, FGD, and KII guides, and standard operating procedures developed for qualitative data collection. IDIs were conducted in Kikuyu, Kiswahili and English depending on the participants’ preferred language. Participants who were fluent in either English or Kiswahili were invited for FGDs that lasted between 60 minutes and 90 minutes; all health care provider interviews were conducted in English. The Kikuyu speaking participants were excluded from FGD because they were too few and since in the FGD the language of discussion is by consensus, it was difficult to get a Kikuyu consensus FGD. Semi-structured interview guides in all the three languages were used to conduct IDIs, FGDs, and KIIs that elicited discussions on attitudes held by the community members towards HIV serodiscordant couples having children.

All interviews were recorded (except one), and the audio recordings in English were transcribed verbatim. Additionally, the audio recordings in Kiswahili and Kikuyu were translated during transcription process by a research assistant with deep understanding of cultural connotations of the study participants. Although back translation was not conducted, a social scientist who was external to the interview team reviewed all the translated transcripts against the recordings. One provider refused audio recording; hand written notes from this interview were used for analysis. A social scientist reviewed all the transcripts by listening to the audio recording for quality control.

### Data analysis

Transcripts were imported into a web-based software program (Dedoose v7.5.9, Socio-Cultural Research Consultants, LLC; Los Angeles, California) and an inductive approach informed by grounded theory methodology was employed to identify community attitudes [27] towards HIV serodiscordant couples having children and accessing safer conception services. Two coders (GT and KD) met on a weekly basis to discuss the codes applied and the differences that arose during coding were resolved in consultation with the study investigators (KN and RH) until consensus was reached. As additional codes emerged, previously coded transcripts were reviewed and the new codes were added. Codes and memos were reviewed and grouped into themes and sub-themes. The coders were coding the transcripts independently and at the 35^th^ transcript i.e. forty percent of the transcripts, the intercoder reliability had been fully achieved.

### Ethics approval and consent to participate

Ethical approval was obtained from the University of Washington Human Subjects Division (#48399) and the Kenya Medical Research Institute Scientific Ethical Review Unit (SSC3003). All participants invited for the IDI, FGD and KII were provided with an information sheet summarizing the study that had been approved by the ethics committee. The interviewer took time to go through the consent form with each participant separately and answered any questions regarding the study. The interviewer emphasized that participation was voluntary and they were free to stop at any point during the interview without any discrimination. To ensure anonymity, the participants were encouraged to use pseudo names rather than their names and avoid stating their HIV status during the interview.

## Results

### Participant characteristics

The median age among members of HIV serodiscordant couples who participated in IDIs and FGDs was 31 and 32 years respectively (interquartile range [IQR] 28-38; 28-35), 11 (44%) participated in the FGDs, and most couples had at least one child. We interviewed 15 HIV positive and 17 HIV negative male and 26 HIV positive and 9 HIV negative female participants. We also conducted 20 key informant interviews with different cadres of health care providers from both private and public health facilities (Table 1).

**Table 1:** Participant characteristics

Members of HIV serodiscordant couples		
Type of Interview	In-depth Interviews (42)	Focus Group Discussions [25]
Age, Median (IQR)	31 (28-38)	32 (28-35)
Female	30 (23-37)	30.5 (27-35)
Male	32 (30-41)	35 (28-42)
No. of participants (Female)	21 (50%)	14 (56%)
No. of participants (Male)	21 (50%)	11 (44%)
Participants HIV status (HIV-positive)	21 (50%)	20 (80%)
Participants HIV status (HIV negative)	21 (50%)	5 (20%)
No. Of children, Median (IQR)	1 (1-2)	1 (0-1)
Providers (participating in key informant interviews) [20]		
Gynecologist	11 (55%)	
Fertility clinics	5 (45.6%)	
Jointly public and private clinics	4 (36.4%)	
Safer conception research sites	2 (18%)	
Nurse counselors	5 (25%)	
Safer conception research sites	3 (60%)	
Public health hospitals	2 (40%)	
Clinical Officers	2 (10%)	
Safer conception research sites	1 (50%)	
Public health hospitals	1 (50%)	
Counselors	2 (10%)	

During data coding and analysis, a number of key themes emerged related to perceptions about pregnancy among people affected by HIV, HIV serodiscordancy, and safer conception services.

### Perceptions about pregnancy among couples living with HIV and discordancy

Participants felt that community attitudes towards HIV serodiscordant couples bearing children were largely negative and presented barriers to accessing safer conception strategies. In particular, participants felt that community members assumed that when conception occurs among HIV serodiscordant couples, sexual and perinatal transmission almost always occurs.

*“Let me say like me now I am positive and my husband is negative now when people see me pregnant if I am carrying the pregnancy now the child I will get they will be afraid of them; they will be saying that they are also positive because I was positive. They don’t usually understand that a woman who is positive can give birth to a child who is not positive.”* (FGD, HIV infected females)

*“They say all of us are positive because they normally don’t understand that someone who is positive cannot be sleeping (having sex) with someone who is negative. They normally don’t understand that there can be positive and negative, now because when they see me pregnant they will know straight (assume) even my husband is positive”* (FGD, HIV infected females*)*

There was a consensus among couples that women are expected to breastfeed their children up to an age of 2 years and those who fail to do that are suspected of being HIV infected. Furthermore, women feared inadvertently disclosing their status when they were seen giving newborns medicine, weaning their infants early, or if the child died immediately after birth.

*“I have seen a girl who was not breastfeeding … and people started saying that, that girl is sick because she … because she was not breastfeeding the child” (*FGD, HIV uninfected males*).*

*“But you hear, you see some get a child and then after a short time the child dies and you hear people saying ‘a certain person is sick, a certain person is not sick’.” (*FGD, HIV uninfected males*).*

*“What they usually say … When this wife gives birth, they want to see if she will breastfeed the child or she will stop and will she give birth to a child who is alive or they will die because many believe if you give birth to a child with virus, they usually die.”* (FGD, HIV infected females*)*

Participants also expressed a community perception that HIV positive parents might not be able to raise a child well and doubt that they would have the strength to provide for young children.

*“What others say is that when she knew she is positive … Why did she carry the pregnancy, she will give birth to a child and how will they be raised, how will they be brought up? Now people usually wonder when they see you, how the child you give birth to will look like, will they be healthy or they will not be healthy … ”* (FGD, HIV infected females)

*I might die and leave them with problems … the way they (children) would have problems.* (IDI, HIV infected female)

Despite perceptions of stigma against HIV serodiscordant couples who bear children, bearing of children by all couples was reported to be a cultural expectation and couples not bearing children were viewed as being incomplete. To satisfy this expectation and desire, couples indicated that they would utilize medically assisted reproduction techniques or adopt children – processes that are not common in Kenya.

*“ … Even if you have a great amount of wealth, but you don’t have a child you are useless. That now is according to our culture”* (FGD, HIV infected males)

*I find that to be good, you know if someone gets their seed (get a child from their sperms) … and even if my seed (sperms) are removed and they are put in her (partner) I will believe that that is my seed (sperm) eeeh, now I am thinking if it can be like that, at least someone has their own child. I think that could be at least good.* (IDI, HIV infected male)

*“ … otherwise even those who are HIV positive would like to have their own children. But they adopt because their fertility has failed, really, not because they are HIV.”* (KII, Health provider, Private fertility clinic)

Although the health care providers rarely raised the issue of childbearing, couples reported feeling stigmatized at public health care facilities and sometimes questioned the advice they received from the health care providers on the issue of pregnancy.

*"But there is one doctor I went to clinic; now you know when you start to wean this one you have to be asked, you ask, now I have to be told now they told me that I am not supposed to get children … I am not supposed to get many children. Would that be true?" (*IDI, HIV infected female)

### Perceptions about safer conception services

By design, we interviewed HIV serodiscordant couples with pregnancy desires, yet in our sample, demand for safer conception services was low and likely stemmed from a lack of knowledge about services and options. Some couples reported that they had never heard of fertility services offered for HIV serodiscordant couples when they are trying to become pregnant.

*… . You know when we knew (HIV status), we did not know that we can get another child now there, there was a little problem but when we were taught, we came here and we were told how we can get another one (child) then we felt that there is no difference, we can look for another one (child).* (IDI, HIV uninfected male)

*“I have never heard even one (fertility clinics), I have not heard in the community of even one. The ones that are usually there; are the ones where someone is positive because they usually go for drugs their services are usually okay … but now for discordant … that one I have never heard, I came to … I knew about discordant but now when I came to learn, I learned from the study.”* (FGD, HIV infected females*)*

A majority of the participants failed to distinguish safer conceptions services from medically assisted reproduction. They, therefore, reported that safer conception services were few and beyond their financial capability. They further noted that it is rare to find the safer conception services being offered in their local health facilities.

*“Let’s just say generally those clinics are rare and if they are there, you find they are private hospitals****.”*** *(*FGD, HIV infected males)

*“Let me say among the rich...They are there but they are usually expensive and most of them are private … eeeh, private and expensive.” (*FGD, HIV infected females)

Most of the couples reported low engagement of male partners in pregnancy planning. Some men further felt that it was only a woman’s responsibility to keep track of her fertility since women directly track their signs of ovulation. *“Me I don’t bother with those days because again, it’s her issue. She is the one who is supposed to know when she is safe and when her fertility is high. She is the one who is supposed to know her calendar so me, (chuckles) I don’t bother so much. That is her responsibility to count days.” (*FGD, HIV infected males)

*“ … She is the one who follows it keenly … even if I said I will follow up; I cannot follow up … That time is when I left that responsibility to her now, for her to count her days well because I do not remember well if it is seven days before or seven days after … .” (*IDI, HIV uninfected male*)*

### Providers’ perceptions towards safer conception services

The health care providers noted that men played an important role in reproduction yet they were not actively participating in seeking safer conception services. Providers expressed their feelings that their efforts to involve men in safer conception seemed futile as male partners would often not attend clinic visits.

*“The male partner involvement has been quite low, it has been quite low so even as you seek these discordant couples it’s not an easy kind of thing because the male involvement is low”. (*KII, Health provider, Fertility clinic-public and private*)*

Providers observed that most HIV serodiscordant couples had low knowledge on safer conception methods and were not sure if they could conceive without infecting the negative partner. Most couples desired support and counseling in order to actualize their desires to conceive while minimizing the risk of sexual transmission.

*" … I mean the question uppermost on the minds of most couples immediately they learn that they are HIV discordant is whether they can be able to have children. And because again of the scarcity of information that is available, most couples feel that it is going to be a very big challenge because of course there is the fear that the uninfected partner is going to get HIV. There is also the fear that the child themselves might also get HIV." (*KII, Health provider, safer conception research site)

However, health care providers reported that they were not adequately prepared or well ‘empowered’ on how to offer safer conception services. They also noted that there were no clear guidelines on how to offer safer conception services to their patients. Therefore, when couples sought safer conception services in their clinics, providers usually referred them to other health facilities.

*"Well, it has been a challenge because most of the time we do not have a structured way of providing solutions to their needs so you have to keep on referring from one point to the other, yeah." (*KII, Health provider, Fertility clinic-public and private*)*

*“ … we try to highlight about it of which even us we are not very much empower … empowered about … sperm washing eeeh so we tell them they are there we refer them … . we can refer them … so we think if those services can be available and we be empowered about the information about it, it can be of great help.”* (KII, Health provider, Public health hospital)

*“This is a young lady, and who is positive, 28 years and eeeh, she really needs … She really want(s) a baby. And she was telling me, ‘what if we take the sperms with a syringe and then we inject … I could see that with sperms now with the syringe and then flush them. I do not know how safe for this thing (sperms) to be alive or they can die on the way and I don't know how … how … effective it can be.” (KII, Health provider, Public health hospital, Thika)*

## Discussion

A key finding from this study is that people affected by HIV perceive negativity in their community towards HIV serodiscordant couples having children and these perceptions pose hurdles for them to access safer conception services. Couples who are suspected to be HIV affected face stigma from community members and pregnancy intensifies the stigma due to the prevailing perception that HIV transmission is unavoidable during the condomless sex that resulted in pregnancy, despite the wide availability and accessibility of HIV treatment and prevention. Although information on safer conception was readily available, couples affected by HIV do not have complete or accurate information about safer conception interventions and the availability of services beyond the research site. Our findings support prior studies in highlighting the missed opportunities to promote safer conception that could be improved by offering couple counseling and tailor-made services for couples in order to encourage male involvement in pregnancy planning and reproductive care and changing the broad perception of fertility services as expensive [21]. Finally, health care providers did not feel well equipped to provide safer conception counseling and offer services, consistent with what has been found in other recent studies [28, 29].

Health care providers rarely brought up the issue of conception among HIV serodiscordant couples, preferring to counsel about contraception and avoiding pregnancy as noted in other sub-Saharan Africa [30, 31]. Our results suggest a need for structured counseling approaches among the health care providers who interact with HIV serodiscordant couples to discuss their fertility goals with them routinely [31, 32]. This may create opportunities for couples to discuss their pregnancy intentions with the health provider in a nonjudgmental environment.

Couples often conflated safer conception with medically assisted reproduction, which is a safer conception option that is associated with higher costs in fertility centers, but not the only option. For this reason, couples associated safer conception with high cost and accessibility only through private health facilities, such as the ones providing fertility care as reported in other studies [2, 33]. In order to minimize the cost of medically assisted services for safer conception, there is need for innovation targeted to low resource settings and promotion of already available fertility measures that can aide in conception. Our finding suggest inclusion of basic fertility screening with low cost ultrasound and sperm counts, teaching couples how to identify peak fertility by tracking ovulation, and offering information on vaginal self-insemination among couples where the female is HIV infected in the public health facilities.

This study was conducted among couples in a research setting who are familiar with ART and PrEP therefore further studies conducted among general population may elicit different perceptions. The findings may not be generalized to represent HIV serodiscordant couples who have not mutually disclosed their status. A majority of the health care providers sampled were from safer conceptions research sites and fertility clinics who were well conversant with safer conception and might not be a reflection of the health care providers in Kenya.

## Conclusions

Negativity towards HIV serodiscordant couples bearing children is still felt among members of HIV serodiscordant partnerships, despite a shift towards HIV becoming more of a chronic illness in sub-Saharan Africa. There is need for health facilities and the community at large to foster an environment that supports pregnancy and embraces opportunities to support HIV serodiscordant couples to achieve their fertility goals. This can be achieved through offering additional training to health care providers on safer conception and sensitizing the community to issues of HIV serodiscordant couples bearing children and the availability of safer conception services for these couples. Current negative attitudes at the community may hinder the use and scale up of safer conception services. Novel interventions and information campaigns are important to change this climate and enable couples to minimize HIV risk during conception.

## Data Availability

Data are available by contacting the International Clinical Research Center at icrc@uw.edu and sending a concept sheet that describes the proposed analysis.

## Abbreviations

ART: Antiretroviral therapy
CCC: Comprehensive care clinics
FGD: Focus group discussion
IDI: In depth interviews
KII: Key informant interviews
PrEP: Pre-Exposure prophylaxis
